# An updated network meta-analysis of EGFR-TKIs and combination therapy in the first-line treatment of advanced EGFR mutation positive non-small cell lung cancer

**DOI:** 10.3389/fonc.2022.616546

**Published:** 2022-08-01

**Authors:** Yuexiao Qi, Xiaojun Xia, Lihua Shao, Liyun Guo, Yumei Dong, Jinhui Tian, Lijun Xu, Ruijun Niu, Shihong Wei

**Affiliations:** ^1^ Department of Radiation Oncology, Gansu Provincial Cancer Hospital, Lanzhou, China; ^2^ Department of Integrated Traditional Chinese Medicine and Western Medicine, Gansu Provincial Cancer Hospital, Lanzhou, China; ^3^ Center of Evidence Based Medicine, Lanzhou University, Lanzhou, China

**Keywords:** non-small-cell Lung cancer, epidermal growth factor receptor tyrosine kinase inhibitors, EGFR-TKIs, anti-angiogenesis, first line, overall survival, network meta-analysis

## Abstract

**Objectives:**

Tyrosine kinase inhibitors (TKIs) are a standard care option in patients with non-small cell lung cancer (NSCLC) with epidermal growth factor receptor (EGFR) mutation. TKI-based combination treatment modes show encouraging outcomes. However, it remains unknown which is the optimal treatment as the first-line regimen for these patients on overall survival (OS).

**Materials and methods:**

Randomized controlled trials and meeting abstracts that investigated EGFR-TKIs alone or in combination as front-line care for patients with NSCLC were systematically searched in relevant databases and reviewed. Fixed and random effects network meta-analysis models were used to estimate progression-free survival (PFS), OS, overall response rate, and grade three and higher adverse events (AEs). Surface under the cumulative ranking curves (SUCRAs) were used to rank treatment effects.

**Results:**

Eighteen studies covering six treatments and involving a total of 4389 patients were included in this network meta-analysis. On OS, the top three treatment were first-generation EGFR-TKIs (1G EGFR-TKIs) plus chemotherapy (SUCRA, 88.1%), osimertinib (SUCRA, 65.8%) and second-generation EGFR-TKIs (2GEGFR-TKIs) (SUCRA, 63.3%). On PFS, the top three treatments were osimertinib (SUCRA, 96.0%), 1G EGFR-TKIs plus chemotherapy (SUCRA, 67.1%), and 1G EGFR-TKIs plus antiangiogenesis (SUCRA, 48.2%). Two types of TKI-based combination therapy have significantly higher risk of grade three and higher AEs than TKI alone.

**Conclusion:**

1G EGFR-TKIs plus chemotherapy and osimertinib seem to be the two better options as first-line care in advanced NSCLC patients with EGFR-mutation. Osimertinib caused the lowest incidence of AEs. However, TKIs-based combination therapy significantly increased AEs.

## Introduction

Lung cancer is the leading cause of cancer-related mortality worldwide (1). Non-small-cell lung cancer (NSCLC) accounts for nearly 85% of all lung cancer cases. Most patients with NSCLC are diagnosed at an advanced stage and have a poor prognosis (2). With the development of new drugs and novel therapeutic strategies, patients with NSCLC harboring epidermal growth factor receptor (EGFR) mutations have prolonged survival and improved prognosis. Since 2004, several important trials have established EGFR tyrosine kinase inhibitor (TKI) therapy as the standard first-line care for patients with EGFR mutations (3–5). First-generation EFGR-TKIs, including gefitinib and erlotinib, improved progression-free survival (PFS) to 9–13.7 months (3–5). Compared with the first-generation EGFR-TKIs, second- and third-generation drugs prolong PFS to 11.0 months (afatinib), 14.7 months (dacomitinib), and 18.9 months (osimertinib), which is significantly better than platinum-based chemotherapy (6–8). Unfortunately, patients with EGFR mutations inevitably develop progression as a result of acquired resistance (3–8), especially among patients with the L858R mutation, who develop resistance earlier than patients with exon 19 deletion.

In order to improve survival, combination therapy strategies are considered and emerging with promising results. The JO25567 trial (JapicCT-111390) identified that the addition of bevacizumab to erlotinib demonstrates significant clinical benefit in improving PFS(16.0 vs. 9.7 months, HR 0.54, 96% CI 0.36–0.79) (9). Similarly, the NEJ009 study (UMIN000006340) shows that concurrent combined treatment of gefitinib and chemotherapy significantly extends both PFS (20.9 vs. 11.9 months, HR 0.49, 95% CI 0.39–0.62) and overall survival (OS) (50.9 vs. 38.8months, HR 0.72, 95% CI 0.55–0.95) compared with EGFR-TKI monotherapy (10). Studies exploring EGFR-TKIs plus the anti-PD-1/PD-L1 antibody in the treatment of EGFR-mutation positive NSCLC are on the way (TATTON, NCT02143466).

Currently, there is a diverse array of treatment strategies under development for metastatic NSCLC (mNSCLC) with sensitizing EGFR mutation. The National Comprehensive Cancer Network and European Society for Medical Oncology (ESMO) guidelines recommend first line osimertinib as the preferred option and other treatment strategies as alternative candidates (11, 12). Also, several previous network meta-analyses compared these multiple treatments in terms of PFS, and the results showed a favorable efficacy of osimertinib compared with other EGFR-TKIs and combination treatments in PFS. As a result, osimertinib is indicated as a preferable option as up-front therapy in patients with activating EGFR mutation mNSCLC (13–15). However, it still remains unclear which treatment showed favorable efficacy in OS and how patients can benefit the most. As the maturity of OS from relevant clinical studies, it is necessary to make a comparison in terms of OS among these available candidates to guide clinicians. This review also aims to develop personalized treatment plans for each patient with activating EGFR mutation NSCLC in an advanced stage by subgroup analysis and provide some valuable clues to guide further studies.

## Materials and methods

The preferred reporting items for systematic reviews and meta-analyses (PRISMA) guidelines and extension for network meta-analysis (16) were strictly followed in this study.

### Literature search strategy

In this network meta-analysis, two authors independently searched PubMed, Web of Science, Embase, MEDLINE, the Cochrane Central Register of Controlled Trials, ClinicalTrial.gov, and the Chinese Biomedical Literature Database (in Chinese) for all studies published before December 31, 2021. The terms used for the search included “non-small-cell lung cancer, NSCLC, erlotinib, gefitinib, icotinib, afatinib, dacomitinib, osimertinib, epidermal growth factor receptor tyrosine kinase inhibitors, EGFR-TKI, anti-angiogenic drugs, bevacizumab, ramucirumab, vascular endothelial growth factor receptor (VEGFR) inhibitors, apatinib and chemotherapy” as well as their synonyms and variations. The full literature search strategy.

In addition, the abstracts from annual meetings and meetings related to lung cancer of the American Society of Clinical Oncology, ESMO, and The World Conference on Lung Cancer were reviewed to identify related studies.

### Study inclusion and exclusion criteria

The inclusion criteria were as follows:

(1). Patients: Patients aged 18 years or older and who were histologically or cytologically confirmed as having NSCLC with clinical stage IIIb or IV harboring EGFR mutation. Patients had no prior antitumor treatment (chemotherapy, radiotherapy, and surgery).
(2). Intervention: 2G EGFR-TKIs (afatinib or dacomitinb) or third-generation EGFR-TKIs (osimertinib) or 1G EGFR-TKIs (erlotinib, gefitinib, and icotinib) plus bevacizumab or ramucirumab or apatinib or plus chemotherapy.
(3). Comparison: the 1G EGFR-TKIs (erlotinib, gefitinib, and icotinib).
(4). Outcome: PFS, OS, objective response rate (ORR), and incidence of adverse events (AEs).
(5). Study design: high-quality randomized controlled trials (RCTs).

Duplication information, animal experimental studies, single-arm clinical trials, retrospective clinical analysis, case reports, and review commentaries were excluded.

### Data extraction and quality assessments

Two reviewers independently assessed each RCT according to the predetermined criteria, and a third reviewer was consulted if there were some disagreements. The same two reviewers independently extracted the data from the selected studies using a standardized data extraction method, including study name, publication year, author information, trial phase, study design, sample size, intervention, primary end points, participant characteristics, response rate, median PFS, median OS, and number of patients who suffered grade three and higher AEs. Hazard ratios (HRs) and 95% confidence intervals (CIs) were directly extracted from qualified trials.

The Cochrane Collaboration Tool was adopted to assess the risk of bias for each RCT, and it is based on various kinds of bias from the following five domains: randomization sequence generation, allocation concealment, blinding of participants and personnel, blinding of outcome assessment, incomplete outcome data, selective reporting, and other biases (17). The quantitative Jadad scale was used to assess study quality (18).

### Statistical analysis

All data analysis is based on the intention-to-treatment principle. The primary outcomes of interest were PFS, OS, ORR, and AEs. For time-to-events variables, PFS and OS were synthesized by HR with corresponding 95% CIs. For dichotomous variables, ORR and AEs were measured by relative risks (RRs) with 95% CIs. A two-tailed *P* value of less than 0.05 was considered statistically significant.

Heterogeneity across studies was evaluated by the Cochran Q total statistic and the inconsistency index (*I^2^* statistic) (19). If *I^2^* > 50% or the *P* value for the *Q* test < 0.1 indicated significant heterogeneity (20), a random effects model was applied to synthesize the available evidence; otherwise, a fixed effects model was used. A sensitivity analysis was also performed to investigate the influence of each single study on the overall estimate size by omitting each one by one if there was significant heterogeneity.

A Bayesian network meta-analysis was performed for all outcome measures in R software (R v4.1.2., https://www.r-project.org) using the package “gemtc” (v1.0-1, https://cran.r-project.org/web/packages/gemtc/index.html), which calls upon JAGS software (v4.3.0., https://mcmc-jags.sourceforge.io) using the rjags package (v4-12, https://cran.r-project.org/web/packages/rjags/index.html) for Markov chain Monte Carlo methods. Cox proportional HRs and their corresponding CIs were used as the summary estimates of relative treatment effects. Log HRs and their corresponding standard errors were used as inputs in the fixed-effect models, which were run with four chains, at least 5000 burns-ins, and 10,000 inferential iterations per chain to ensure model convergence. All analyses were replicated in WinBUGS software (version 1.4.3) for comparative validation in R software in order to double-check the results.

Rank probabilities for each treatment were also produced on Bayesian NMA by calculating the probability of each treatment that could achieve the best rank among the included treatments (21). Surface under the cumulative ranking curves (SUCRAs) were calculated to rank probabilities of all treatments in R software (R v4.1.2.). Each statistical test was considered two-sided.

## Results

### Search results and study selection

**Figure 1:**
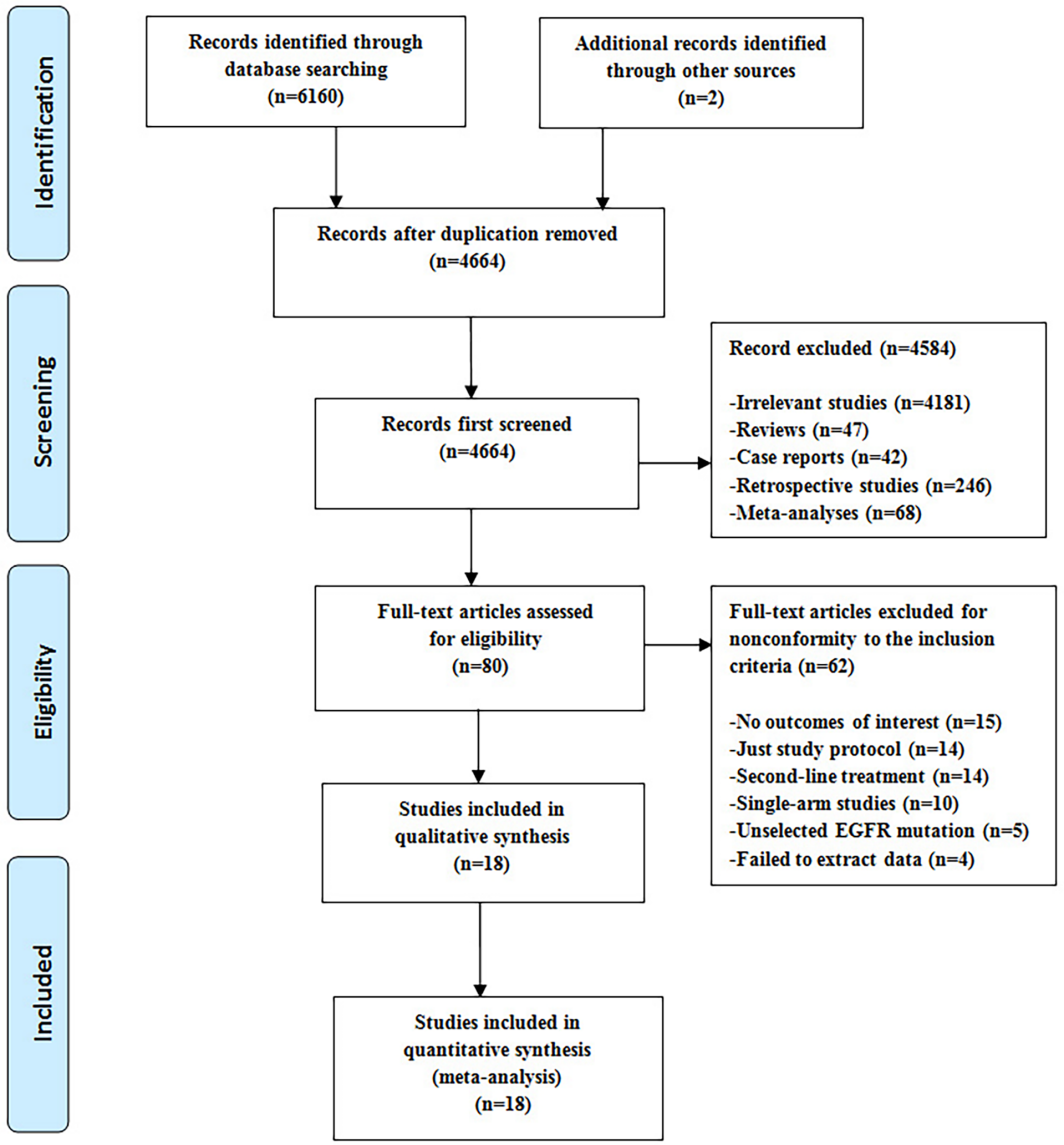
Search strategy and follow chat of the included studies.

As shown in **Figure 1**, after reviewing abstracts and titles, 80 potentially eligible studies were assessed carefully by full-text review. Among them, 62 studies were excluded for the following reasons: 15 studies lacked outcomes of interest, 14 studies were just trial protocols (study designs) without study results, 14 studies referred to second-line treatments; 10 studies were single-arm studies, five trials included patients without selecting EGFR mutation, and four trials failed to extract data. Finally, 18 RCTs involving 4389 participates were considered to meet the inclusion criteria and included in the network meta-analysis to compare five treatments, including the 1G EGFR-TKIs, 2G EGFR-TKIs, third-generation EGFR-TKIs (3G-EGFR-TKIs), and the 1G EGFR-TKIs plus chemotherapy or plus antiangiogenic drugs (6–10, 22–39). Among these 18 trials, 17 were reported as publications (6–10, 22–39), and some data of interest in one study was extracted from a meeting abstract (10). Two RCTs compared afatinib or dacomitinib with gefitinib, respectively (7, 8, 36, 37), one RCT compared osimertinib with erlotinib or gefitinib (6, 38), eight RCTs compared erlotinib or gefitinib plus chemotherapy with erlotinib or gefitinib alone (10, 29–35). Six RCTs compared erlotinib plus bevacizumab or ramucirumab with erlotinib alone (9, 22–27). One RCT compared gefitinib plus apatinib, a VEGFR 2 TKI, with gefitinib alone (28). One RCT compared high-dose icotinib with routine-dose icotinib in patients with the L858R mutation (39).

### Population characteristics

**Figure 2:**
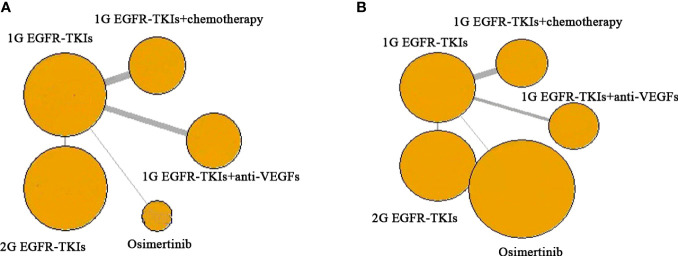
Network of the comparisons for the network meta-analysis. **(A)** PFS; **(B)** OS. Each circular node represents a type of treatment. The circle size is proportional to the total number of studies. The width of lines is proportional to the number of studies performing a head-to-head comparison in the same study. Abbreviations: First-generation EGFR-TKIs (1G EGFR-TKIs); Second-generation EGFR-TKIs (2G EGFR-TKIs); anti-vascular endothelial growth factors drugs (anti-VEGFs).

In each trial, the demographic characteristic of participates were generally well-balanced between different trial arms, within each trial, and across trials. The sample size of included studies ranged from 50 to 556. The basic characteristics of the included 18 RCTs are summarized in **Table 1**. Median age ranged from 55 to 67.5 years. Most of the patients were in stage IIIb and IV of the disease. Exon 19 deletion and exon 21-L858R were mainly EGFR mutations. The majority of the included trials in two combination treatment divisions and the INCREASE trial were conducted in Asia (9, 10, 22–24, 26, 30–35, 39). A graphic network structure shows the network of trials for PFS and OS (**Figure 2**). Each circle node represents a special type of treatment. Direct comparisons are represented by the black lines connecting treatments. The width of lines is proportionate to the number of studies that perform head-to-head comparisons in the same study (40) (**Figure 2**).

**Table 1:** Characteristics of the included randomized trials in the meta-analysis.

Study	Region	Phase	Treatment	Sample size (no.)	No. Of EGFR mutation		Efficacy			Grade≥3 AEs (%)
					ex19del	L858R	ORR (%)	PFS (months)	OS (months)	
**JO25567 (** 9, 22)	Japan	II	Erlotinib+bevacizumab	75	40	35	69	16.0	47.0	91
*(JapicCTI-111390)*	multicenter		Erlotinib	77	40	37	64	9.7	47.4	53
**NEJ026 (** 23, 24)	Japan	III	Erlotinib+bevacizumab	112	56	56	81	16.9	NA	98
(*UMIN000017069)*	multicenter		Erlotinib	112	55	57	74	13.3	NA	46
**Stinchcombe et al (** 25)	USA	II	Erlotinib+bevacizumab	43	29	14	81	17.9	32.4	NR
*(NCT01532089)*	multicenter		Erlotinib	45	30	15	83	13.5	50.6	NR
**CTONG1509 (** 26)	China	III	Erlotinib+bevacizumab	157	82	75	86.3	18.0	NR	53.5
*(NCT02759614)*	multicenter		Erlotinib	154	79	75	87.4	11.3	NR	25.5
**RELAY (** 27)	worldwide	III	Erlotinib+ramucirumab	224	123	99	76	19.4	NR	72
*(NCT02411448)*	multicenter		Erlotinib+placebo	225	120	105	75	12.4	NR	54
**CTONG1706 (** 28)	China	III	Gefitinib+Apatinib	157	81	74	77.1	13.7	NR	84.1
*(NCT02824458)*	multicenter		Gefitinib+placebo	156	83	73	73.7	10.2	NR	37.7
**CALGB30406 (** 29)	USA	II	Paclitaxel+carboplatin+erlotinib	33	16	17	73	17.2	38.1	NA
*(NCT00126581)*			Erlotinib	33	23	10	70	14.1	31.3	NA
**Yang et al. (** 30)	East Asia	III	Pemetrexed+cisplatin+gefitinib	26	14	10	65.4	12.9	32.4	34
*(NCT01017874)*			Gefitinib	24	11	13	70.8	16.6	45.7	16
**Cheng et al. (** 31)	East Asia	II	Pemetrexed+gefitinib	126	65	52	80.2	15.8	43.4	53
*(NCT01469000)*			Gefitinib	65	40	23	73.8	10.9	36.8	12
**An et al. (** 32)	China	II	Pemetrexed+gefitinib	45	16	29	80.0	18.0	34.0	NR
			Gefitinib	45	17	28	73.3	14.0	32.0	NR
**Han et al. (** 33)	China	II	Pemetrexed+carboplatin+gefitinib	40	21	19	82.5	17.5	32.6	NR
*(NCT02148380)*			Gefitinib	41	21	20	65.9	11.9	25.8	NR
**Noronha (** 34)	India	III	Pemetrexed+carboplatin+gefitinib	174	107	60	84	20.9	50.9	65.3
*(CTRI/2016/08/007149)*			Gefitinib	176	109	60	67	11.9	38.8	31.0
**NEJ009 (** 10)	Japan	III	Pemetrexed+carboplatin+gefitinib	170	93	69	75.3	20.9	50.9	75
*(UMIN000006340)*			Gefitinib	172	95	67	68.3	11.9	38.8	49.4
**Xu et al. (** 35)	China	II	Pemetrexed+carboplatin+icotinib	90	51	38	77.8	16.0	36.0	NR
*(NCT02031601)*			Icotinib	89	52	37	64.0	10.0	34.0	NR
**LUX-Lung7 (** 7, 36)	Worldwide	II	Afatinib	160	93	67	70.0	11.0	27.9	31.0
*(NCT01024413)*	multicenter		Gefitinib	159	93	66	56.0	10.9	24.5	18.0
**ARCHER1050 (** 8, 37)	Japan,Korea	III	Dacomitinib	227	134	93	75.0	14.7	34.1	63
*(NCT01774721)*	multicenter		Gefitinib	225	133	92	72.0	9.2	26.8	41
**FLAURA** (6, 38)	Worldwide	III	Osimertinib	279	158	97	80.0	18.9	38.6	32.0
*(NCT02296125)*	multicenter		Gefitinib/erlotinib	277	155	90	76.0	10.2	31.8	41.0
**INCREASE (** 39)	China	II	Icotinib high dose	90	0	90	73.0	12.9	6.67	NR
*(NCT02404675)*	multicenter		Icotinib routine dose	86	0	86	48.0	9.2	8.20	NR

NA, not available; Outcomes: progression-free survival (PFS); objective response rate (ORR); adverse events (AEs); overall survival (OS). NR, not reach.

### Quality assessment and publication bias

All 18 included trials were judged to have low risk of bias through using the risk of bias tool described in the Cochrane Handbook for Systematic Reviews of Interventions (17). All included trials generated an adequate randomization sequence without observable allocation concealment and selective outcome reporting.

### Overall survival

There were 15 trials contributing to network meta-analysis for OS. The 2G EGFR-TKIs (HR 0.81, 95%CI 0.67–0.98), 1G EGFR-TKIs plus chemotherapy (HR 0.73, 95%CI 0.63–0.85), and osimertinib (HR 0.80, 95%CI 0.64–1.00) were all more effective in comparison with 1G EGFR-TKIs in improving OS except 1G EGFR-TKIs plus anti-VEGF drugs (HR 0.95, 95%CI 0.78–1.20). Osimertinib was not clearly superior to 2G EGFR-TKIs (HR 0.99, 95%CI 0.74–1.30), 1G EGFR-TKIs plus anti-VEGF drugs (HR 0.84, 95%CI 0.63–1.10) or plus chemotherapy (HR 1.10, 95%CI 0.84–1.40). According to SUCRAs, the rank probability of OS was as follows: 1G EGFR-TKIs plus chemotherapy (88.1%) > osimertinib (65.8%) > 2G EGFR-TKIs (63.3%) > 1G EGFR-TKIs plus anti-VEGF agents (24.5%) > 1G EGFR-TKIs (8.3%).

**Figure 3:**
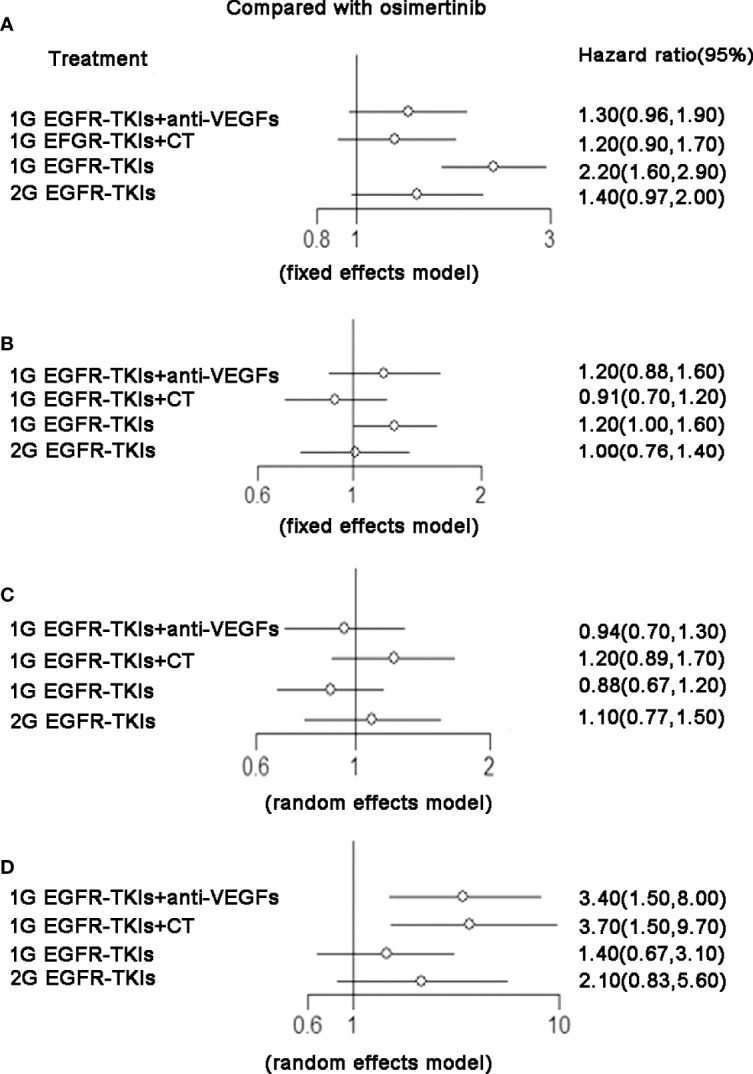
**(A)** PFS, forest plot of hazard ratio (HRs) for progression-free survival; **(B)** OS, forest plot of hazard ratio (HRs) for overall survival;**(C)** ORR, forest plot of hazard ratio (HRs) for objective response rate; **(D)** SAE, forest plot of hazard ratio (HRs) for Serious Adverse Events. Results were based on fixed effects or random effects method. First-generation EGFR-TKIs (1G EGFR-TKIs); Second-generation EGFR-TKIs (2G EGFR-TKIs); anti-vascular endothelial growth factor (anti-VEGF).

There were nine trials that reported OS and corresponding HRs in patients with specific mutations. For patients with the ex19del mutation, osimertinib (HR 0.80, 95%CI 0.64–1.00), 2G EGFR-TKIs (HR 0.81, 95%CI 0.67–0.98), and 1G EGFR-TKIs plus chemotherapy (HR 0.73, 95%CI 0.63–0.85) were all more effective in comparison with 1G EGFR-TKIs monotherapy in improving OS. Osimertinib was not clearly superior to 2G EGFR-TKIs (HR 0.99, 95%CI 0.74–1.30), 1G EGFR-TKIs plus anti-VEGF agents (HR 0.84, 95%CI 0.63–1.10), or plus chemotherapy (HR 1.10, 95%CI 0.84–1.40). 1G EGFR-TKIs plus anti-VEGF agents did not improve OS (HR 0.95, 95%CI 0.78–1.20) compared with 1G EGFR-TKIs. According to SUCRAs, 1G EGFR-TKIs plus chemotherapy (86.7%), osimertinib (80.7%), and 2G EGFR-TKIs (47.5%) were the top three treatments in terms of OS for patients with the ex19del mutation (**Figure 3** and **Table 2**). For patients with the L858R mutation, 13 trials with five treatments reported OS and contributed to the meta-analysis of OS. Only 1G EGFR-TKIs plus chemotherapy tended to improve OS (HR 0.71, 95%CI 0.50–1.00) in comparison with 1G EGFR-TKIs. However, osimertinib was not clearly superior to 2G EGFR-TKIs (HR 1.20, 95%CI 0.80–1.90), 1G EGFR-TKIs plus anti-VEGF agents (HR 1.10, 95%CI 0.69–1.90), and 1G EGFR-TKIs plus chemotherapy (HR 1.40, 95%CI 0.87–2.30). According to SUCRAs, 1G EGFR-TKIs plus chemotherapy (84.6%), 2G EGFR-TKIs (67.9%), and 1G EGFR-TKIs plus anti-VEGF agents (50.5%) were the top three treatments in terms of OS for patients with the L858R mutation (**Figure 3** and **Table 2**).

**Table 2:** Results of network meta-analysis for PFS and OS.

a. Hazard ratios (HR) with 95% confidence interval (CI) for progress-free survival (PFS) in patients with ex19del.									
**Osimertinib**									
0.67 (0.41, 1.10)		**2G-TKIs**							
0.70 (0.45, 1.10)		1.00 (0.72, 1.50)		**1G-TKIs+anti-VEGFs**					
0.78 (0.49, 1.20)		1.20 (0.80, 1.70)		1.10 (0.81, 1.50)		**1G-TKIs+CT**			
**0.43 (0.29, 0.64)**		**0.64 (0.48, 0.86)**		**0.62 (0.49, 0.77)**		**0.55 (0.44, 0.69)**		**1G-TKIs**	
b. Hazard ratios(HR) with 95% confidence interval(CI) for overall survival (OS) in patients with ex19del.									
**Osimertinib**									
0.99(0.74, 1.30)		**2G-TKIs**							
0.84(0.63, 1.10)		0.85(0.65, 1.10)		**1G-TKIs+anti-VEGFs**					
1.10(0.84, 1.40)		1.10(0.87, 1.40)		1.30(1.00, 1.70)		**1G-TKIs+CT**			
**0.80(0.64, 1.00)**		**0.81(0.67, 0.98)**		0.95(0.78, 1.20)		**0.73(0.63, 0.85)**		**1G-TKIs**	
c.Hazard ratios (HR) with 95% confidence interval(CI) for progress-free survival(PFS) in patients with L858R.									
**Osimertinib**									
0.77(0.50, 1.20)	**2G-TKIs**								
0.80(0.55, 1.20)	1.00(0.76, 1.40)		**1G-TKIs+anti-VEGFs**						
0.97(0.65, 1.50)	1.30(0.90, 1.80)		1.20(0.92, 1.60)		**1G-TKIs+CT**				
0.68(0.42, 1.10)	0.89(0.58, 1.40)		0.85(0.58, 1.20)		**0.70(0.47, 1.00)**		**High 1G-TKIs**		
**0.51(0.36, 0.72)**	**0.66(0.51, 0.86)**		**0.64(0.54, 0.76)**		**0.53(0.42, 0.65)**		0.75(0.53,1.10)		**1G -TKIs**
d.Hazard ratios (HR) with 95% confidence interval(CI) for overall survival (OS) in patients with L858R.									
**Osimertinib**									
1.20(0.80, 1.90)	**2G-TKIs**								
1.10(0.69, 1.90)	0.92(0.58, 1.40)		**1G-TKIs+anti-VEGFs**						
1.40(0.87, 2.30)	1.10(0.72, 1.80)		1.20(0.74, 2.00)		**1G-TKIs+CT**				
1.00(0.71, 1.40)	0.80(0.61, 1.10)		0.88(0.61, 1.30)		**0.71(0.50, 1.00)**		**1G -TKIs**		

OS, overall survival; PFS, progression-free survival; ORR, objective response rate. First-generation EGFR-TKIs (1G-TKIs); Second-generation EGFR-TKIs (2G-TKIs); anti-vascular endothelial growth factor (anti-VEGF), Chemotherapy (CT). Significant hazard ratios are in bold.

Exploration of OS in potential subgroups of interest (based on the existence of CNS metastasis, gender, and ECOG PS) are calculated but that of other interests (based on age, ethnicity, and smoking status) was not feasible due to inconsistent reporting of group data across the trials. In subgroup analysis, two combination treatments, 1G EGFR-TKIs plus chemotherapy (HR 0.57, 95%CI 0.36–0.9, SUCRA 85.6%) and plus antiangiogenic drugs (HR 0.62, 95%CI 0.38–1.00, SUCRA 77.9%) showed a significant improvement of OS in patients with CNS metastasis compared with 1G EGFR-TKIs alone. They were ranked the top two treatments for patients with brain metastasis. Better efficacy of osimertinib was observed in the female group (HR 0.79, 95%CI 0.60–1.04, SUCRA 73.4%) as well as 1G EGFR-TKIs plus chemotherapy (HR 0.66, 95%CI 0.44–0.99, SUCRA 75.9%) and osimertinib (HR 0.70, 95%CI 0.54–0.91, SUCRA 69.3%) in the ECOG PS 1 group.

### Progress-free survival

**Figure 4:**
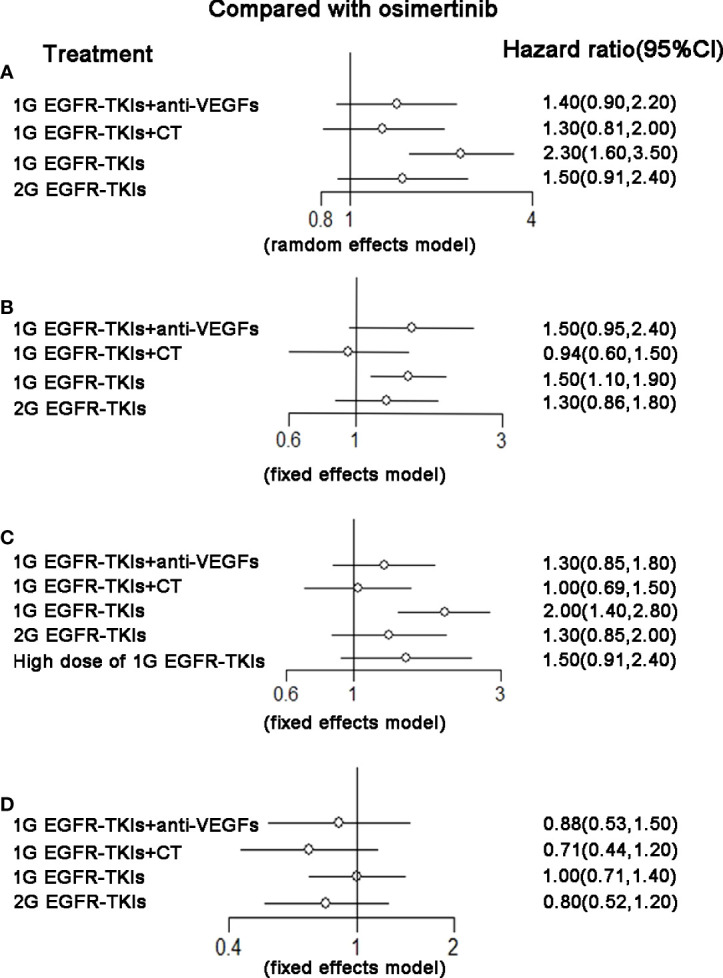
**(A)** Forest plot of hazard ratio (HRs) for progression-free survival (PFS) in patients with 19 deletion mutation; **(B)** Forest plot of hazard ratio (HRs) for progression-free survival (PFS) in patients with L858R mutation; **(C)** Forest plot of hazard ratio (HRs) for overall survival (OS) in patients with 19 deletion mutation; **(D)** Forest plot of hazard ratio (HRs) for overall survival (OS) in patients with L858R mutation. Results were based on fixed effects or random effects methods. First-generation EGFR-TKIs (1G EGFR-TKIs); Second-generation EGFR-TKIs (2G EGFR-TKIs); anti-vascular endothelial growth factor drugs (anti-VEGFs.).

There were 18 trials contributing to the network meta-analysis for PFS analysis. As shown in **Figure 4** and **Table 3**, comparing the five treatments, osimertinib (HR 0.43, 95%CI 0.29–0.64), 2G EGFR-TKIs (HR 0.64, 95%CI 0.48–0.86), 1G EGFR-TKIs plus anti-VEGF agents (HR 0.62, 95%CI 0.49–0.77), and 1G EGFR-TKIs plus chemotherapy (HR 0.55, 95%CI 0.44–0.69) were all more effective in comparison with 1G EGFR-TKI monotherapy in improving PFS. Osimertinib was clearly superior to 2G EGFR-TKIs (HR 0.71, 95%CI 0.54–0.93) and 1G EGFR plus anti-VEGF agents (HR 0.75, 95%CI 0.53–1.00), but it was not more effective than 1G EGFR-TKIs plus chemotherapy (HR 0.81, 95%CI 0.57–1.10). According the SUCRAs, the rank probability of PFS was as follows: osimertinib (96.0%) > 1G EGFR-TKIs plus chemotherapy (67.1%) > 1G EGFR-TKIs plus anti-VEGF agents (48.2%) > 2G EGFR-TKIs (38.7%) > 1G EGFR-TKIs (0.03%).

**Table 3:** Results of network meta-analysis for PFS, OS, ORR and SAEs.

a. Hazard ratios(HR) with 95% confidence interval(CI) for progress-free survival (PFS)				
**Osimertinib**				
**0.71 (0.54, 0.93)**	**2G-TKIs**			
**0.75 (0.53, 1.00)**	1.10 (0.80, 1.40)	**1G-TKIs+anti-VEGFs**		
0.81 (0.57, 1.10)	1.10 (0.86, 1.50)	1.10 (0.86, 1.30)	**1G-TKIs+CT**	
**0.46 (0.34, 0.62)**	**0.65 (0.52, 0.81)**	**0.62 (0.53, 0.72)**	**0.57 (0.49, 0.67)**	**1G-TKIs**
b. Hazard ratios(HR) with 95% confidence (CI) for overall survival (OS).				
**Osimertinib**				
0.99 (0.74, 1.30)	**2G-TKIs**			
0.84 (0.63, 1.10)	0.85 (0.65, 1.10)	**1G-TKIs+anti-VEGFs**		
1.10 (0.84, 1.40)	1.10 (0.87, 1.41)	**1.30 (1.00, 1.70)**	**1G-TKIs+CT**	
**0.80 (0.64, 1.00)**	**0.81 (0.67, 0.98)**	0.95 (0.78, 1.20)	**0.73 (0.63, 0.85)**	**1G-TKIs**
c.Odds ratios(OR) with 95% confidence interval(CI) for objective response (ORR).				
**Osimertinib**				
0.92 (0.65, 1.30)	**2G-TKIs**			
1.10 (0.78, 1.40)	1.20 (0.88, 1.50)	**1G-TKIs+anti-VEGFs**		
0.82 (0.60, 1.10)	0.89 (0.69, 1.20)	**0.78 (0.63, 0.96)**	**1G-TKIs+CT**	
1.10 (0.87, 1.50)	**1.20 (1.00, 1.50)**	1.10 (0.93, 1.20)	**1.40 (1.20, 1.60)**	**1G-TKIs**
c.Odds ratios(OR) with 95% confidence interval(CI) for serious adverse events (SAEs).				
**Osimertinib**				
0.47 (0.18, 1.20)	**2G-TKIs**			
**0.29 (0.12, 0.66)**	0.63 (0.32, 1.20)	**1G-TKIs+anti-VEGFs**		
**0.27 (0.10, 0.65)**	0.58 (0.26, 1.20)	0.93 (0.47, 1.70)	**1G-TKIs+CT**	
0.69 (0.32, 1.50)	1.50 (0.84, 2.70)	**2.40 (1.70, 3.40)**	**2.50 (1.60, 4.60)**	**1G-TKIs**

OS, overall survival; PFS, progression-free survival; ORR, objective response rate. SAEs, serious adverse events; First-generation EGFR-TKIs (1G-TKIs); Second-generation EGFR-TKIs (2G-TKIs); anti-vascular endothelial growth factor (anti-VEGF). Significant hazard ratios are in bold

There were 13 trials that reported HRs in patients with specific mutations, 2284 (52.0%) patients had an ex19del mutation, and 1892 (39.8%) had an L858R mutation. For patients with the ex19del mutation, osimertinib (HR 0.43, 95%CI 0.29–0.64), 2G EGFR-TKIs (HR 0.64, 95%CI 0.48–0.86), 1G EGFR-TKIs plus anti-VEGF agents (HR 0.62, 95%CI 0.49–0.77), and 1G EGFR-TKIs plus chemotherapy (HR 0.55, 95%CI 0.44–0.69) were all more effective in comparison with 1G EGFR-TKI monotherapy in improving PFS. Osimertinib was not clearly superior to 2G EGFR-TKIs (HR 0.67, 95%CI 0.41–1.10), 1G EGFR-TKIs plus anti-VEGF agents (HR 0.70, 95%CI 0.45–1.10), or 1G EGFR-TKIs plus chemotherapy (HR 0.78, 95%CI 0.49–1.20). According to SUCRAs, the top three treatments were osimertinib (94.2%), 1G EGFR-TKIs plus chemotherapy (67.6%), and the 1G EGFR-TKIs plus anti-VEGF agents (46.8%) in terms of PFS. For patients with the L858R mutation, in addition to the above 13 trials, there was a special treatment reported by a trial for patients with the L858R mutation, which increased the dose of incotinib, a kind of first-generation EGFR-TKI, to improve the efficacy. All 14 trials with six treatments were included in the network meta-analysis for PFS analysis. Osimertinib (HR 0.51, 95%CI 0.36–0.72), 2G EGFR-TKIs (HR 0.66, 95%CI 0.51–0.86), 1G EGFR-TKIs plus anti-VEGF agents (HR 0.64, 95%CI 0.54–0.76), and 1G EGFR-TKIs plus chemotherapy (HR 0.53, 95%CI 0.42–0.65) were all more effective in comparison with 1G EGFR-TKI monotherapy with routine dosage in improving PFS. No treatment was clearly superior to others among the four treatments. However, a high dose of 1G EGFR-TKIs (HR 0.75, 95%CI 0.53–1.10) was not more effective than the normal dose of 1G EGFR-TKIs. According to the SUCRAs, osimertinib (85.3%), 1G EGFR-TKIs plus chemotherapy (84.7%), and 1G EGFR-TKIs plus anti-VEGF agents (52.3%) were the top three in terms of PFS (**Figure 3** and **Table 2**).

### Objective response rate

For network meta-analysis of ORR, there were 17 trials that covered five treatments included. As shown in **Figure 4** and **Table 3**, 1G EGFR-TKIs plus chemotherapy was considered the highest probability of being the best treatment to achieve a response (92.3%), followed by 2G EGFR-TKIs (68.4%), osimertinib (47.3%), 1G EGFR-TKIs plus anti-VEGF drugs (33.3%) and 1G EGFR-TKIs (8.7%).

### Serious Adverse Events (SAEs)

As shown in **Figure 4** and **Table 3**, regarding grade three or worse AEs, compared with osimertinib, 1G EGFR-TKIs plus anti-VEGF drugs (HR 2.40, 95%CI 1.70–3.40) and 1G EGFR-TKIs plus chemotherapy (HR 2.50, 95%CI 1.60–4.60) led to a significantly higher risk of grade three and worse AEs. Both 1G EGFR-TKIs plus anti-VEGF drugs and 1G EGFR-TKIs plus chemotherapy have a significantly higher risk of grade three and worse AEs than 1G EGFR-TKIs alone. But there were no significant differences between these two kinds of combined therapies (HR 0.93, 95%CI 0.47–1.70). According to SUCRAs, osimertinib had the lowest risk of grade three and worse AEs and the rank probability was as follows: osimertinib (96.1%) > 1G EGFR-TKIs (76.7%) > 2G EGFR-TKIs (42.7%) > 1G EGFR-TKIs plus anti-VEGF agents (22.5%) > 1G EGFR-TKIs plus chemotherapy (12.2%).

## Discussion

In patients with advanced EGFR-mutant NSCLC, EGFR-TKIs are approved as first-line options because all of them show superior efficacy and prolonged PFS compared with platinum-based chemotherapy (3–8). The second-generation TKIs (afatinib and dacomitinib) and third-generation TKIs (osimertinib) were more effective in comparison with first-generation TKIs at improving PFS (7–9) in the first-line setting. Survival of advanced NSCLC with activating EGFR-mutation is significantly improved due to the introduction of osimertinib. The FLAURA trial (NCT02296125) demonstrated that osimertinib significantly extended the mPFS (18.9 months) compared with the first-generation EGFR-TKIs (gefitinib or erlotinib, 10.2 months) (7). In combined treatment strategies, both the addition of chemotherapy or anti-angiogensis to 1G EGFR-TKIs demonstrate considerable clinical benefit with improved PFS (9, 10, 23–35). The precise network meta-analysis demonstrated first-line osimertinib is superior to 1G and 2G EGFR-TKIs as well as the combination of anti-VEGF agents and 1G EGFR-TKIs and ranked top in terms of PFS (13–15). The results of our study are consistent with these previous meta-analyses. In the FLAURA study (NCT02296125), first-line osimertinib also has significant OS improvement compared with 1G EGFR-TKIs, which established the foundation of osimertinib as the standard first-line care in advanced NSCLC with activating EGFR-mutations (38). The AURA3 study (NCT02151981) demonstrated that 2L osimertinib exceeded mPFS (10.1 vs. 4.4 months; HR 0.30, 95%CI 0.23–0.41) compared with chemotherapy in patients with T790M mutations followed by 1G/2G EGFR-TKIs as 1L therapy, which established osimertinib as the standard of care for patients who develop a T790M mutation after 1G/2G EGFR-TKI therapy as a first line (41). There is a concern raised as to which setting of osimertinib is most beneficial as the lL or 2L therapy. Some clinicians may worry that, if osimertinib is set in the first line, there are no targeted drugs available in the 2L treatment after osimertinib resistance. In fact, if osimertinib was reserved in 2L therapy, a portion of patients have a probability to not be tested for and found to be positive for T790M mutation and lose the opportunity to accept osimertinib therapy. Also, not all patients develop a resistance mechanism to the T790M mutation after earlier generation EGFR-TKI therapy, and some patients do not survive to accept 2L therapy. A real-world study shows that only 72% of patients were tested for the T790M mutation after 1G/2G EGFR-TKI resistance, and the remaining nearly 30% of patients were untested. About half of the tested patients were T790M-positive. Only one third of the patients received osimertinib upon progression on 1G/2G EGFR-TKIs (42). Moreover, the FLAURA trial demonstrated that a significant OS improvement with osimertinib in the 1L setting exists in spite of the fact that 47% of patients assigned to division of first line 1G/2G EGFR-TKIs received osimertinib as the second line therapy (38). Therefore, setting osimertinib as the first-line treatment seems to be more favored. Further trials need to provide more evidence to determine which line osimertinib set in is more efficient and rational. The APPLE study (NCT02856893), an ongoing phase II trial, was designed to evaluate the best strategy for sequencing gefitinib and osimertinib in patients with an EGFR mutation and EGFR TKI treatment-naive advanced NSCLC in 1L treatment, which could help to determine when osimertinib is most beneficial as 1L or 2L treatment (43).

OS is considered the gold standard for choosing the optimal therapy. As far as we are aware, this study is the first network meta-analysis to compare the mature OS of these multiple treatments. Results show the combined treatments of 1G EGFR-TKIs and chemotherapy surpassed osimertinib and was ranked the top in terms of OS in both all population and patients with CNS metastasis. It indicates that combination therapy with osimertinib and chemotherapeutic drugs seems to be a promising strategy to further improve survival and even to approach a cure. However, a randomized phase 2 clinical trial (jRCTs071180062) showed that, as a second-line therapy after initial EGFR-TKI resistance, the addition of carboplatin-pemetrexed to osimertinib failed to improve PFS (14.6 vs. 15.8 months; HR 1.09, 95%CI 0.51–2.32) and OS (HR 2.42, 95%CI 0.82–7.15) compared with standard osimertinib monotherapy (44). Outcomes of ongoing FLAURA 2 (NCT04035486), a phase 3 clinical trial, evaluate osimertinib and platinum-pemetrexed versus osimertinib in treatment-naive advanced NSCLC patients with EGFR-mutation, are eagerly awaited to assess whether this combination confers a significant survival benefit in a first line setting.

The EGFR and VEGF pathways share downstream signaling targets, and dual blockade of EGFR and angiogenic caused synergetic effects (45). Clinically, the addition of bevacizumab and remucirumab to 1G EGFR-TKIs significantly improved PFS in advanced NSCLC with EGFR mutation (10, 22–28). In a first line setting, the combination of erlotinib and bevacizumab demonstrates an improved PFS of 16.0, 16.9, and 18.0 months in JO25567 (JapicCTI-111390), NEJ026 (UMIN000017069) and CTONG1509 (NCT02759614) trials, respectively (9, 23–28). But the significant PFS benefit observed with erlotinib plus bevacizumab failed to translate into a significant OS benefit (22, 24, 26). The combination of erlotinib and ramucirumab showed a significantly improved PFS of 19.4 months in the RELAY trial, and the OS remains immature. In 2L treatment, both the WJOG 8715L (UMIN000023761) and BOOSTER (NCT03133546) trials demonstrate the addition of bevacizumab to osimertinib in advanced NSCLC patients with the EGFR mutation and acquired T790M mutation after failure of 1L EGFR-TKI treatment was not associated with an improvement in both PFS and OS, which suggests this combination strategy may not be able to increase efficacy over osimertinib monotherapy (46, 47). Outcomes of ongoing studies in EGFR-TKI naive patients accepting osimertinib plus bevacizumab (NCT4181060) or ramucirumab (NCT03909334) may further examine the role of an antiangiogenic-included combination strategy in 1L treatment.

Ex19del and L858R are two of the most common types of EGFR mutations, but they have biological differences and specific mechanisms that account for their different efficacy to treatment (48). Subgroup analyses of major studies reveal a tendency for patients with ex19del to benefit more from treatment with three generations of EGFR-TKI candidates than patients with L858R. Taking into account the subgroup analysis in each landmark trial, patients with both ex19del and L858R could significantly benefit from treatment of afatinib, dacomitinib, and osimertinib compared with first-generation EGFR-TKIs in terms of PFS (3–8). However, only osimertinib improved the OS of patients with the ex19del mutation (HR 0.68, 95%CI 0.51–0.90) (38). No significant OS benefit from treatment with second-generation EGFR-TKIs (afatinib, HR 0.80, 95%CI 0.64–1.00; dacomitinib, HR 0.80, 95%CI 0.64–1.00) and even osimertinib (HR 1.00, 95%CI 0.71–1.40) was observed in the subgroup of patients with the L858R mutation (36–38). The INCREASE trial (NCT02404675), a randomized phase II trial, demonstrated high-dose icotinib improved PFS in comparison with routine-dose icotinib in mNSCLC patients harboring the L858R mutation (HR 0.75, 95%CI 0.53–1.05) (39). In combination treatments, NEJ009 (UMIN000006340) showed significant improvements in PFS from a combination of EGFR-TKIs and chemotherapy for patients harboring both the ex19del mutation (HR 0.47, 95%CI 0.34–0.64) and the L858R mutation (HR 0.55, 95%CI 0.38–0.80) in IL treatment, but subgroup data on OS are not available (10). A number of meta-analyses offer strong evidence that patients with both ex19del (HR 0.61, 95%CI 0.49–0.75, *p* = 0.00) and patients with L858R (HR 0.59, 95%CI 0.47–0.73, *p* = 0.00) benefit from a combination of elortinib and antiangiogenesis therapy on PFS (49, 50). In the CTONG1509 trial (NCT02759614), the PFS of patients with the L858R mutation achieved 19.5 months in the combination group, which is the best PFS observed to date (26). The result was approximately double that of the erlotinib-alone group (9.7 months) and even exceeded the 14.4 months PFS of patients receiving osimertinib, which is followed by erlotinib and ramucirumab (19.4 months) in the RELAY (NCT02411448) trial and erlotinib and bevacizumab (17.4 months) in NEJ026 (6, 23, 27). The data suggest that patients with L858R derive more benefit from the addition of an anti-angiogensis to erlotinib. Unfortunately, this significant prolonged PFS did not translate into a significant OS benefit in patients with the L858R mutation in both NEJ026 and CTONG1509, and OS data are awaited from the RELAY trial to further evaluate the role of this combination strategy for patients with the L858R mutation (24, 26). A group of prospective trials focuses on the combination of osimertinib and anti-angiogenic drugs (UMIN000028071, NCT 0281579) is expected to further improve the efficacy and break though the treatment bottleneck of patients with L858R mutation in the first line setting.

EGFR-TKIs remains the standard care of advanced NSCLC patients with sensitizing EGFR mutations. The molecular mechanism of acquired resistance in up-front treatments are of great importance because choosing the optimal subsequent therapies after disease progression on 1L therapy depends largely on the mechanisms driving resistance. T790M mutation is the most common resistance mechanism to 1G and 2G EGFR-TKIs, occurring in up to two thirds of patients and for whom osimertinib is the standard of care (51). In the NEJ026 and JO25567 studies, the frequency of T790M mutation in progression patients after 1L treatments was similar between the bevacizumab plus erlotinib and erlotinib alone groups, which identified that the combination of bevacizumab and erlotinib had no effect on the acquired T790M mutation, which allowed patients in both groups to have same chance to use osimertinib in a second line setting (9, 22–24). For patients who are T790M mutation-negative, there is a lack of effective options in the second line setting and where there remains an urgent unmet medical need. Continuing with EGFR-TKIs, local therapy and systemic chemotherapy are current alternative options, and clinical determination depends on patients’ characteristics. Current explorations cover bevacizumab plus chemotherapy and atezolizumab plus bevacizumab plus chemotherapy for these T790M-negative patients after 1G/2G EGFR-TKI treatment (51, 52).

The molecular mechanisms of resistance to osimertinib are complex and still under study. Patterns of molecular resistance vary depending on whether osimertinib is given in a first line setting or in a subsequent line. It seems that the resistance mechanism spectrum of osimertinib in the second line is more complex than that in in the first line setting (53). However, the resistance mechanism of osimertinib in both clinical contexts could be grouped into two categories: on-target EGFR-dependent and off-target EGFR-independent mechanisms (54). EGFR-dependent resistance typically is related to alterations in the banding site caused by additional EGFR-mutations, which disrupt the osimertinib binding. The most common EGFR-dependent resistance mutation of osimertinib is the EGFR exon 20 C797S mutation, and other EGFR alterations include C797X, L718O, and S768I in the front line and T790M absence, L792H/L792V, G796S/G796C, and G724S in the second line (53–55). EGFR-independent mechanisms are mostly associated with aberrant downstream signaling or alternative pathway activation and histological transformations. MET amplification is the most frequent off-target mechanism of resistance to osimertinib, which activates the MET-related downstream PI3K/AKT and MAPK pathways. Other mechanisms include HER2 amplification and the emergence of NRAS, PI3KCA, BRAF, and KRAS mutations (56). Currently, platinum-based combination chemotherapy, platinum plus pemetrexed in most cases, is approved as the standard of care in patients after osimertinib resistance. For patients with transformation to SCLC and squamous cell carcinoma, treatments preferred are platinum-etoposide and platinum-gemcitabine, respectively. A treatment strategy of combined MET and EGFR inhibition in the setting of MET amplification–driven osimertinib resistance seems a promising and compelling approach in preliminary results of the INSIGHT 1 trial (NCT01982955) assessing the combination of tepotinib and gefitinib and in the CHRYSALIS-1 study (NCT02609776) evaluating lazertinib, a 3G EGFR-TKI, in combination with amivantamab, which is a special antibody that can inhibit both EGFR and MET receptors (57, 58). As with the MET amplification, a combination of EGFR-TKIs and an inhibitor of the acquired mutation is an emerging trend in the treatment strategy for patients with acquired HER2, ALK, RET, BRAF, and other oncogenes. Brigatinib plus cetuximab could be of benefit and may be potentially effective to improve outcomes in patients with acquired co-mutations in C797S and EGFR T790M–driven resistance (59). The prospective ELIOS trial (NCT03239340) will provide a more complete picture of osimertinib resistance in the 1L setting and help to develop a more reasonable treatment strategy for sequential treatment.

Several potential limitations should be considered when interpreting the results of this study. First, heterogeneity exists in network meta-analyses, especially in subgroup analyses. The main intrinsic sources of heterogeneity were from different trial designs, including different treatments, races, and designs. It was difficult to resolve even using the individual patient data. Second, one study was only presented as abstract, which led to insufficient data in subgroups being available. This limitation built a barrier to reach a definitive conclusion about the superiority between different treatments. Finally, most of the included RCTs in the EGFR-TKIs plus chemotherapy group (30–35) and EGFR-TKIs plus anti-angiogenesis group (9, 22–24, 26, 28) were performed in Asian countries; therefore, the vast majority of participants were Asians. And data on other races were not available.

## Conclusions and perspectives

In summary, our study is, to our knowledge, the first network meta-analysis to estimate and compare the mature OS of five treatments as the first-line treatment in advanced NSCLC patients who are EGFR mutation-sensitive. IG EGFR-TKIs plus chemotherapy and osimertinib had high SUCRAs for PFS and OS and ranked as the top two best treatments. With regard to AEs, osimertinib had an obvious advantage due to a significantly low risk of SAEs. However, limitations of the study, including a single RCT investigating osimertinib and lacking data on the combination regimens from other races than Asian. Further investigations and updated analyses are needed to provide additional evidence to verify the most favorable first-line management in patients harboring activated EGFR-mutated NSCLC. From our perspective, further direction of effort includes next-generation EGFR-TKIs, the resistance mechanisms of EGFR-TKIs and new agents to target these resistances, novel combination modes, and control of AEs.

## Data availability statement

All data generated or analyzed in this study are included in this article/**Supplementary Material**. Further enquires can be directed to the corresponding authors.

## Author contributions

YQ and SW designed and conceived the study. LG, LX and YD collected the data. LS analyze the data and performed the statistical analysis. Prof. JT gave the important guidance for statistical analysis and methodology.XX and RN provided critical intellectual contributions. And YQ drafted the manuscript. All authors reviewed and approved the final version.

## Funding

This study was supported by the Special Project for Major Disease Prevention and Treatment of Administration of Traditional Chinese Medicine in Gansu Province (grant number GZKZD-2018-03), the Health Industry Scientific Research Program of Gansu Province in 2019 (grant number GSWSKY-2019-82), and the Science and Technology Development Guiding Program of Lanzhou City of Gansu Province (grant number 2019-ZD-134).

## Acknowledgments

The authors would like to thank JT for his advice and assistance in data statistical analysis, and thank Dr. Shun Li for language editing.

## Conflict of interest

The authors declare that the research was conducted in the absence of any commercial or financial relationships that could be construed as a potential conflict of interest.

## Publisher’s note

All claims expressed in this article are solely those of the authors and do not necessarily represent those of their affiliated organizations, or those of the publisher, the editors and the reviewers. Any product that may be evaluated in this article, or claim that may be made by its manufacturer, is not guaranteed or endorsed by the publisher.

## Abbreviations

NSCLC, non-small cell lung cancer; RCTs, randomizesponded controlled trials; OS, overall survival; PFS, progression-free survival; ORR, objective response rate; AEs, adverse effects; HR, hazard ratio; RR, relative risk; CI, confidence intervals; FDA, Food and Drug Administration; VEGF, vascular endothelial growth factor; EGFR-TKI, epidermal growth factor receptor tyrosine kinase inhibitor; PRISMA, Preferred Reporting Items for Systematic Reviews and Meta-Analysis statement; ECOG, Eastern Cooperative Oncology Group; NCCN, National Comprehensive Cancer Network; ESMO, European Society for Medical Oncology; WCLC, The World Conference on Lung Cancer; SUCRAs, Surface under the cumulative ranking curves; 1G EGFR-TKI, first-generation EGFR-TKI; 2G EGFR-TKI, second-generation EGFR-TKI; 1L, first line; 2L, second line.
