# Supplementary material for: An updated network meta-analysis of EGFR-TKIs and combination therapy in the first-line treatment of advanced EGFR mutation positive non-small cell lung cancer
Source: Front Oncol. 2022 Aug 1;12:616546. doi: 10.3389/fonc.2022.616546 (PMC9376288; doi:10.3389/fonc.2022.616546)
Supplement: Supplementary file 1 [file Table_1.docx]

**Supplemental Table 1**  Search strategy

| # | Query |
| --- | --- |
| #1 | “Lung Neoplasms”[MeSH] |
| #2 | Lung Neoplasms[tiab] OR Neoplasms, Lung[tiab] OR Lung Neoplasm[tiab] OR Neoplasm, Lung[tiab] OR Neoplasms, Pulmonary[tiab] OR Neoplasm, Pulmonary[tiab] OR Pulmonary Neoplasm[tiab] OR Pulmonary Neoplasms[tiab] OR Lung Cancer[tiab] OR Cancer, Lung[tiab] OR Cancers, Lung[tiab] OR Lung Cancers[tiab] OR Pulmonary Cancer[tiab] OR Cancer, Pulmonary[tiab] OR Cancers, Pulmonary[tiab] OR Pulmonary Cancers[tiab] OR Cancer of the Lung[tiab] OR Cancer of Lung[tiab] |
| #3 | "Carcinoma, Non-Small-Cell Lung"[MeSH] |
| #4 | Carcinoma, Non Small Cell Lung[tiab] OR Carcinomas, Non-Small-Cell Lung[tiab] OR Lung Carcinoma, Non-Small-Cell[tiab] OR Lung Carcinomas, Non-Small-Cell[tiab] OR Non-Small-Cell Lung Carcinomas[tiab] OR Nonsmall Cell Lung Cancer[tiab] OR Non-Small-Cell Lung Carcinoma[tiab] OR Non Small Cell Lung Carcinoma[tiab] OR Carcinoma, Non-Small Cell Lung[tiab] OR Non-Small Cell Lung Cancer[tiab] OR NSCLC[tiab] |
| #5 | #1 OR #2 OR #3 OR #4 |
| #6 | Advanced[tiab] OR Stage IV[tiab] OR Stage 4[tiab] OR Stage four[tiab] OR StageIIIB[tiab] OR Metastatic[tiab] OR Metastases[tiab] |
| #7  #8 | "ErbB Receptors"[MeSH]  ErbB Receptors[tiab] OR Epidermal Growth Factor Receptor Kinase[tiab] OR Epidermal Growth Factor Receptor Protein-Tyrosine Kinase[tiab] OR Epidermal Growth Factor Receptor Protein Tyrosine Kinase[tiab] OR Epidermal Growth Factor[tiab] OR Receptor, ErbB-1[tiab] OR ErbB-1 Receptor[tiab] OR Receptor, ErbB 1[Title/Abstract] OR Epidermal Growth Factor Receptor[tiab] OR Receptor*, EGF[tiab] OR EGF Receptor*[tiab] OR Receptor*, Epidermal Growth Factor[tiab] OR EGR[tiab]) OR EGFR[tiab] OR ErbB‐1[tiab] OR HER1[tiab] OR EGFR TKI[tiab] ORKinase Inhibitor[tiab] OR Kinase Inhibitors[tiab] OR Gefitinib[tiab] OR Erlotinib[tiab] OR Icotinib[tiab] OR Afatinib[tiab] OR Dacomitinib[tiab] OR Osimertinib[tiab] OR Rociletinib[tiab] OR Naquotinib[tiab] OR Nazartinib[tiab] OR Abivertinib[tiab] OR Mavelertinib[tiab] OR Alflutinib[tiab] OR Olafertinib[tiab] OR Lazertinib[tiab] OR Almonertinib[tiab] OR Rezivertinib[tiab] OR Almonertinib[tiab] OR TAS-121[tiab] |
| #9 | Chemotherapies[tiab] OR Chemotherapy[tiab] OR Docetaxel[tiab] OR Pemetrexed[tiab] OR Gemcitabine[tiab] OR Vinorelbine[tiab] OR Paclitaxel[tiab] OR |
| #10  #11 | "Angiogenesis Inhibitors"[MeSH]  Angiogenesis Inhibitors[tiab] OR Neovascularization Inhibitors[tiab] OR Angiogen* Antagonists[tiab] OR Angiogen* Inhibitors[tiab] OR Angiostatic Agents[tiab] OR Agents, Angiostatic[tiab] OR Antagonists, Angiogen*[tiab] OR Anti-Angiogenetic Agents[tiab] OR Agents, Anti-Angiogenetic[tiab] OR Anti Angiogenetic Agents[tiab] OR Anti-Angiogenic Drugs[tiab] OR Anti Angiogenic Drugs[tiab] OR Drugs, Anti-Angiogenic[tiab] OR Antiangiogenic Agents[tiab] OR Agents, Antiangiogenic[tiab] OR Inhibitors, Angiogen*[tiab] OR Inhibitors, Angiogenesis Factor[tiab] OR Inhibitors, Neovascularization[tiab] OR Angiogenesis Factor Inhibitors[tiab] OR Factor Inhibitors, Angiogenesis[tiab] OR bevacizumab[tiab] OR Anlotinib[tiab] OR Ramucirumab[tiab] OR Endostatin*[tiab] OR Sorafenib[tiab] OR Sunitinib[tiab] OR Pazopanib[tiab] OR Vandetanib[tiab] OR Axitinib[tiab] OR Regorafenib[tiab] OR Lenvatinib[tiab] OR Apatinib[tiab] OR Anlotinib[tiab] |
| #12 | #7 OR #8 OR #9 OR #10 OR #11 |
| #13 | First-line[tiab] OR Untreated[tiab] OR Chemotherapy naïve[tiab] OR Frontline[tiab] OR Treatment naïve[tiab] |
| #14 | Randomized Controlled Tial[pt] |
| #15 | Controlled Cinical Trial[pt] |
| #16 | Randomized[tiab] |
| #17 | Placebo[tiab] |
| #18 | Randomly[tiab] |
| #19  #20 | Trial[tiab]  Drug Therapy[sh] |
| #21 | Groups[tiab] |
| #22 | #14 OR #15 OR #16 OR #17 OR #18 OR #19 OR #20 OR #21 |
| #23 | Animals[mh] |
| #24 | Humans[mh] |
| #25 | #23 NOT #24 |
| #26 | #22 NOT #25 |
| #27 | #5 AND #6 AND #12 AND #13 AND #22 AND #26 |
